# Biosynthesis of selenium nanoparticles by *Azoarcus* sp. CIB

**DOI:** 10.1186/s12934-016-0510-y

**Published:** 2016-06-14

**Authors:** Helga Fernández-Llamosas, Laura Castro, María Luisa Blázquez, Eduardo Díaz, Manuel Carmona

**Affiliations:** Environmental Biology Department, Centro de Investigaciones Biológicas-CSIC, Ramiro de Maeztu 9, 28040 Madrid, Spain; Material Science and Metallurgical Engineering Department, Facultad de Químicas, Universidad Complutense de Madrid, Madrid, Spain

**Keywords:** Nanoparticles, Selenium, Bioremediation, *Azoarcus*, Rice, Nanotechnology, Biotechnology

## Abstract

**Background:**

Different bacteria have been reported so far that link selenite resistance to the production of metallic selenium nanoparticles (SeNPs). Although SeNPs have many biotechnological applications in diverse areas, the molecular mechanisms involved in their microbial genesis are not fully understood. The *Azoarcus* genus is a physiologically versatile group of beta-proteobacteria of great environmental relevance. *Azoarcus* sp. CIB is a facultative anaerobe that combines the ability to degrade under aerobic and/or anaerobic conditions a wide range of aromatic compounds, including some toxic hydrocarbons such as toluene and *m*-xylene, with an endophytic life style in the root of rice. We unravel here an additional physiological feature of the strain CIB that is related to its resistance to selenium oxyanions and the formation of SeNPs.

**Results:**

This work is the first report of a member of the *Azoarcus* genus that is able to anaerobically grow in the presence of selenite. Electron microscopy preparations and X-ray spectroscopy analyses demonstrate the reduction of selenite to spherical electron-dense SeNPs whose average size was 123 ± 35 nm of diameter. Our data suggest that the main molecular mechanism of selenite resistance resides on an energy-dependent selenite exporter. *Azoarcus* cells trigger the synthesis of SeNPs when they reach the stationary-phase of growth, and either the exhaustion of electron donor or acceptor, both of which lead to starvation conditions, produce the reduction of selenite to red elemental selenium. *Azoarcus* becomes a promising biocatalyst, either as whole cells or cellular extracts, for the anaerobic and/or aerobic green synthesis of SeNPs.

**Conclusions:**

*Azoarcus* turns out to be a new eco-friendly system to reduce selenite and produce spherical SeNPs. Moreover, this is the first report of a rice endophyte able to produce SeNPs. Since *Azoarcus* is also able to degrade both aerobically and anaerobically toxic aromatic compounds of great environmental concern, it becomes a suitable candidate for a more sustainable agricultural practice and for bioremediation strategies.

**Electronic supplementary material:**

The online version of this article (doi:10.1186/s12934-016-0510-y) contains supplementary material, which is available to authorized users.

## Background

The ability to resist heavy metal and metalloids toxicity is widely spread in prokaryotes since bacteria have been exposed to metals from the beginning of the life [1]. The molecular mechanisms underlying the metal resistance have been extensively reviewed over the past 30 years [2–4]. In particular, bacterial resistance to chalcogens such as selenium has been paid attention in the last years [5, 6]. The distribution of the different species of selenium may vary in the environment depending on the prevailing redox conditions [6]. The two more abundant oxyanions of selenium are the selenate [Se(VI), SeO_4_^2−^] and selenite [Se(IV), SeO_3_^2−^] and both can be biologically converted to the less toxic insoluble mineral form Se (0). The molecular basis of biological selenite reduction to Se (0) has not been completely elucidated. Selenite is presumed to be uptaken by the cells via a sulfate transporter and is reduced to elemental selenium, which is endowed with a characteristic red color, in the cytoplasm most probably by thiol-mediated reduction [6]. Some bacteria link their resistance to selenium oxyanions to the production of selenium nanoparticles (SeNPs) with defined size and shape [7]. The synthesis of metallic SeNPs could be intracellular and/or extracellular depending on the mechanism involved. Thus, extracellular accumulation of SeNPs appears to be linked directly to anaerobic respiration whereas the intracellular accumulation might be the result of detoxification of selenite [6].

Despite selenium was long regarded as a toxin [8], at the same time is an essential trace element with importance in several physiological functions, such as biosynthesis of selenocysteine, coenzyme Q, glutathione peroxidase or thioredoxin reductase [9, 10]. Selenium supplementation in the diet has been associated with a health benefits effect [11, 12]. SeNPs also have semiconductor and photoelectrical properties and they have been used successfully in applications ranging from solar cells, photographic exposure meters, photocopiers and rectifiers [13, 14]. SeNPs have also applications in diverse areas such as electronics, cosmetics, coatings, packaging, biotechnology and biomedicine [15]. It has been found that SeNPs exhibit low cytotoxicity compared with selenium compounds and posses excellent anticancer and therapeutic activities making them apt for medical applications [8, 16]. SeNPs have anticancer activity against kidney, breast, lung and osteosarcoma [17, 18] and hence, can be used as chemopreventive and chemotherapeutic agents. On the other hand, SeNPs synthesized from a biological source possess a significant antimicrobial activity against pathogenic bacteria, fungi and yeast [19]. The physical/chemical methods to produce monodispersed SeNPs with different size and shape are extensively applied, but the presence of toxic chemicals limits their application in clinical fields and cause major concerns [20]. Understanding the molecular mechanisms underlying the bacterial production of chalcogen nanostructures is gaining increased interest in the field of nanotechnology, with the potential for exploitation in bionanomaterial fabrication [21], however there are still unresolved questions on the microbial genesis and secretion of the SeNPs [6].

*Azoarcus* sp. CIB is a facultative anaerobic β-Proteobacterium with the ability to degrade under aerobic and/or anaerobic conditions a wide range of aromatic compounds including some toxic hydrocarbons such as toluene and *m*-xylene [22–24]. In addition, it has been recently demonstrated that CIB strain is able to grow in association with plants, living as an endophyte in the root of the rice [25]. Moreover, genome mining in CIB suggested the existence of a high number of gene clusters encoding potential resistance to heavy metals [26]. The results presented in this work demonstrate that *Azoarcus* sp. CIB is the first *Azoarcus* strain described so far with significant resistance to selenium oxyanions and able to produce SeNPs. Since the strain CIB is able to degrade toxic aromatic compounds, interact with plants, and resist some metals and metalloids, the use of this bacterium could offer an efficient, economic and sustainable bioremediation technology. The use of CIB as an eco-friendly system to obtain SeNPs with benefits to human health and some other biotechnological applications is also discussed.

## Results and discussion

### Selenite resistance in *Azoarcus* sp. CIB

**Fig. 1:**
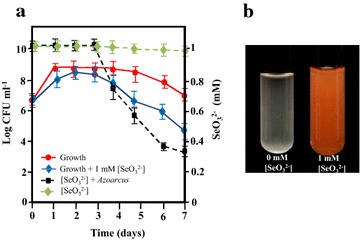
Time course of anaerobic growth and selenite reduction by *Azoarcus* sp. CIB. **a** Time course of the anaerobic growth of *Azoarcus* cells and kinetics of selenite removal. *Error bars* represent standard deviations of at least three independent experiments. **b** Images of cultures of *Azoarcus* anaerobically grown for 5 days

To analyze the possibility of extending the abilities of CIB for bioremediation strategies, we studied the level of resistance of the strain CIB to selenite compounds. To do that, we tested the resistance/sensibility of the strain CIB to the oxyanion selenite (Na_2_SeO_3_). Whereas the strain CIB was able to anaerobically grow in the presence of up to 8 mM selenite, a lower selenite resistance level (1 mM) was observed in aerobic conditions (Additional file 1: Figure S1). The level of resistance to selenite in *Azoarcus* is similar to that reported in other bacteria, e.g. *Bacillus subtilis* (>1 mM), *Rhodospirillum rubrum* DSM467 (>2 mM), *Wolinella succinogenes* DSM 1740 (1 mM) or *R. metallireducens* (6 mM), but lower than that the one reported in highly resistant bacteria such as *Pseudomonas* sp. strain CA5 (>150 mM) or *Rhizobium leguminosarum* bv*.viceae* (200 mM) [27]. The viability of *Azoarcus* in the presence of 1 mM of selenite was followed during 7 days of growth (Fig. 1). After 72 h, the culture turned into an orange color solution (Fig. 1b), suggesting the reduction of selenite to elemental selenium [28] and the cell viability decreased by more than 3 orders of magnitude, indicating cellular lysis (Fig. 1a) that was confirmed by phase contrast microscopy analyses. When the colored cultures were pelleted by centrifugation, the liquid supernatant remained uncolored and the pellet was bright red. ICP-OES analyses revealed that the disappearance of the selenite ions occurred during the stationary-phase of cellular growth (Fig. 1a). The participation of the bacteria in the selenite reduction was required since no change in selenite concentration or appearance of orange color was observed when cells were not present in the culture (Fig. 1).

It has been described that some bacteria are able to methylate selenium oxyanions as part of their resistance mechanisms generating organic methylated forms, such dimethyl selenide or dimethyl diselenide, that share a garlic acid-like smell [29]. Remarkably, a clear garlic acid-like smell was associated to *Azoarcus* cells cultured anaerobically in the presence of selenite, thus suggesting that the CIB strain might be also able to produce/generate organic volatile forms of selenium as an alternative resistance strategy. A control of selenite reduction and production of volatile selenium compounds by stationary-phase regulatory proteins has been previously suggested in other bacteria such *R. rubrum* [30] and *Rhodopseudomonas palustris* strain N [31].

Taken together all these results reveal that strain CIB is the first example of a member of the *Azoarcus* genus, a physiologically versatile group of bacteria some of which are of great biotechnological interest [26], that is able to anaerobically grow in the presence of selenite, hence opening new avenues for its application in bioremediation strategies.

### Production of selenium nanoparticles by *Azoarcus* sp. CIB

**Fig. 2:**
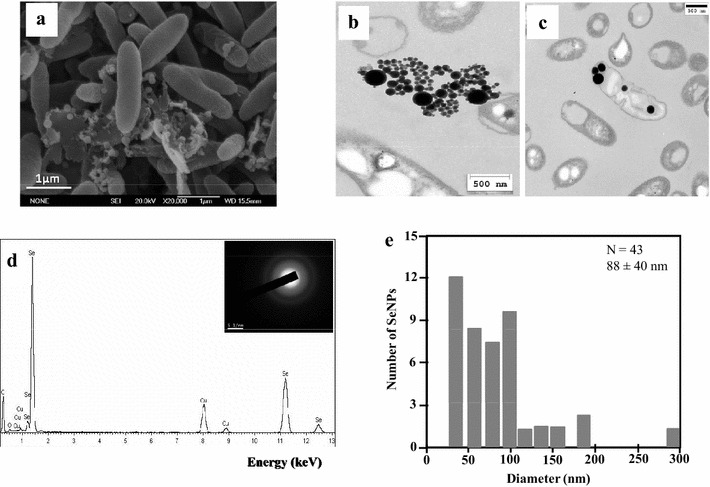
Analysis of the SeNPs production by stationary-phase *Azoarcus* sp. CIB cells. SEM analysis (**a**) and TEM analysis showing nanoparticles outside (**b**) or inside (**c**) the cells. *Black line* on **c** represents 500 nm. **d** EDX analysis of one SeNP of panel **a**. In the *inset* are shown the diffuse rings in the SAED (selected area electron diffraction) pattern of one SeNP. White line on the inset represents 5 1/nm. **e** Size distribution of SeNPs produced by *Azoarcus*

As described above, *Azoarcus* is able to resist selenite. Since several bacteria are able to link the reduction of the metal oxyanions to the production of metallic nanoparticles [7], we analyzed here if strain CIB was able to associate selenite reduction to the production of SeNPs. To this end, when the selenite-containing culture medium turned into an intense orange color (5 days of growth) we collected the cells by centrifugation and observed them by using scanning electron microscopy (SEM). The nanoparticles were located outside the cells and sometimes appeared attached to cell debris, most probably as a result of cell lysis (Fig. 2a). The association of SeNPs suggests that they were not actively exported to the medium, as it was proposed in *Enterobacter cloacae* SLD1 a-1 [32], but rather released after cell death and bacterial lysis, as it was suggested in other bacteria [33]. Other mechanisms proposed for exporting the intracellular synthesized SeNPs are those involving the SefA protein that binds to and stabilizes the nanoparticles in *Thauera selenatis* [34], and the formation of membrane vesicles in *R. rubrum* [35].

The use of Transmission electron microscopy (TEM) showed that most of the SeNPs were localized outside the cells and only few inside the bacteria (Fig. 2b, c), with spherical shape and average size 88 ± 40 nm (Fig. 2e). The elemental analysis using energy-dispersive X-ray spectroscopy (EDX) showed that the electron-dense particles presented the specific selenium absorption peak (Fig. 2d). The diffuse rings in the SAED (selected area electron diffraction) pattern suggested that selenium is present in its amorphous form (Fig. 2d, inset).

### Insights into the molecular mechanism of selenite resistance during the exponential growth of *Azoarcus* sp. CIB

**Fig. 3:**
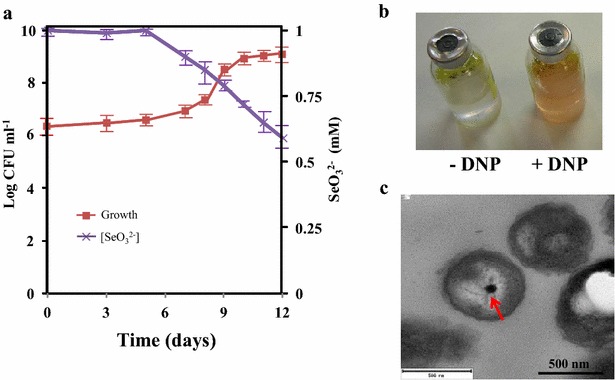
Selenite reduction by *Azoarcus* cells growing in the presence of DNP. **a** Time course of the anaerobic growth of *Azoarcus* and selenite removal from the growth medium supplemented with DNP. **b** Cultures flasks of CIB cells grown without DNP (-DNP) or supplemented with DNP (+DNP) at the exponential phase of growth after 24 h or 7 days of incubation, respectively. **c** TEM (thin section) of *Azoarcus* cultures collected at the exponential phase of growth. The *red arrow* indicates the presence of a SeNP deposited in the bacterial cytoplasm

Selenite consumption and formation of SeNPs in anaerobic cultures of *Azoarcus* was observed only at the stationary-phase of growth (Fig. 1a), suggesting that during exponential growth phase the bacteria are able to resist selenite by using a different detoxification mechanism. It has been postulated that in *R. rubrum* selenite may be kept outside the cell until the end of exponential phase because the bacteria are equipped with a specific transport system [36]. A similar selenite resistance mechanism could be also present in CIB. According to our results, we hypothesized that this selenite export system was energy-dependent and only when cells were highly energized, i.e. at the exponential growth phase, selenite could be secreted out of the cell. On the contrary, energy depleted cells at the stationary-phase allowed that selenite could be maintained enough time in the cytoplasm and become reduced to metallic SeNPs. To experimentally substantiate this hypothesis, we studied the selenite reduction in cells partially depleted of intracellular energy by addition to the culture medium of non-lethal concentrations of the uncoupling agent 2,4-dinitrophenol (DNP). DNP dissipates the electrochemical gradient across the cytoplasmic membrane, thereby blocking the subsequent ATP synthesis [37]. Thus, the addition of DNP to exponentially grown CIB cultures would mimic the expected reduction of intracellular energy at the stationary-phase and the export of selenite out of the cell would then be decreased. *Azoarcus* cells grown in the presence of selenite and DNP removed selenite from the culture medium (Fig. 3a) and accumulated SeNPs (Fig. 3c) during the exponential growth phase, in contrast to what has been observed in the absence of DNP (Fig. 1). Accordingly, the culture medium of *Azoarcus* cells grown in the presence of selenite and non-lethal DNP concentrations presented a distinctive orange color at the exponential phase, whereas cultures grown in the absence of DNP remained uncolored (Fig. 3b).

All these data allow us to speculate that during the exponential growth phase of *Azoarcus* the main molecular mechanism of selenite resistance is an energy-dependent selenite exporter, which remains to be identified, that avoids the accumulation of selenite inside the cells. Interestingly, this mechanism of selenite resistance appears to be functional only under anaerobic conditions but not when the cells are in the presence of oxygen, that avoids growth at selenite concentrations higher than 1 mM.

### Studies on the molecular basis of selenite reduction in *Azoarcus* sp. CIB

Since knowledge of enzymatic basis of microbial selenite reduction may be used to enhance selenite bioremediation capability in genetically-engineered bacteria or in cell-free enzyme systems [38], we investigated the molecular basis of SeNPs formation in CIB. Possible mechanisms of selenite reduction include simple chemical reduction induced by specific medium components or reduction induced by the action of bacterial enzymes [35]. As indicated above, no change of selenite concentration was observed in sterile control medium confirming the participation of the bacteria in the selenite reduction (Fig. 1a). After addition to an *Azoarcus* cell culture of a solution of 1 mM selenite and incubation at 30 °C for 24 h, the supernatants (heated and unheated) and thermally treated cells were not capable of reducing selenite to Se (0). However, the unheated crude cell extract produced intense orange coloration (Additional file 2: Figure S2). These data are consistent with the participation of an enzyme or any other thermo labile cell component in selenite reduction to elemental Se in *Azoarcus* cells.

So far, a number of bacterial enzymes have been suggested to play a role in selenite reduction. It has been speculated that a periplasmic respiratory nitrite reductase was responsible for selenite reduction. Two non-homologous enzymes, the cytochrome *d1*-containing NirS and the copper-type NirK nitrite reductases, catalyze the reduction of nitrite to nitric oxide [39, 40]. The copper-containing nitrite reductase NirK from *Rhizobium sullae* [41] has been involved in selenite reduction, but the role of nitrite reductase NirS from *T. selenatis* in selenite reduction has not been confirmed yet [34]. *Azoarcus* sp. CIB lacks an ortholog of the *nirK* gene but its genome contains a *nirS* ortholog (*AzCIB_3601*) [26]. However, the participation of NirS in selenium reduction requires further investigation.

Glutathione is the primary reduced thiol in *Escherichia coli*, and it was proposed as one of the best candidates for bacterial intracellular selenite reduction [35]. The main source of reduced glutathione in the cell is the glutathione reductase (GOR), but we could not identify any *gor* gene ortholog in the genome of CIB [26]. Moreover, the addition of 3 mM buthionine sulfoximide (BSO), a compound that has been shown to decrease the intracellular glutathione concentration [42], to the *Azoarcus* cells growing anaerobically in the presence of 1 mM selenite did not decrease the formation of SeNPs with respect to that observed when the cells grew in the absence of BSO (Additional file 3: Figure S3). These results suggest that selenite reduction in CIB does not depend on levels of intracellular glutathione.

**Fig. 4:**
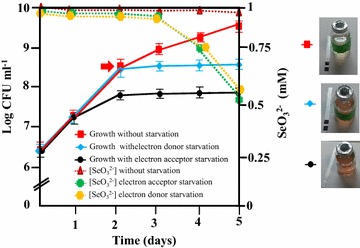
Starvation conditions are needed for SeNPs biosynthesis with *Azoarcus* cells. *Error bars* represent standard deviations of at least three independent experiments

The synthesis of SeNPs only when the *Azoarcus* cultures reached the stationary-phase (Fig. 1) suggests that depletion of an important resource needed for growth might be responsible of the formation of such nanoparticles. To investigate further this observation, we analyzed whether the starvation of the carbon source or that of the electron acceptor, both of which lead the cultures to a stationary-phase, may generate differences in selenite reduction. To do that, we anaerobically grew *Azoarcus* cells under, (i) carbon source starvation conditions, or (ii) electron acceptor starvation conditions. In both cases, it was possible to observe the presence of orange coloration in the culture medium at 3 days, when cells reached the stationary-growth phase (Fig. 4). Interestingly, by preventing the starvation conditions, we could consequently avoid the formation of metallic Se (Fig. 4). Taken together all these results show that starvation is needed for selenite reduction and that either the lack of electron donor or electron acceptor, both of which trigger the entrance in the stationary-phase, produce the reduction of the selenite to red elemental selenium in *Azoarcus*. Although the cellular component(s) involved in selenite reduction is still unknown, it might be the result of the action of unspecific oxidoreductases present in the CIB cells, and further work should be performed to clarify this issue.

### Exploring additional *Azoarcus* sp. CIB-based systems for SeNPs bioproduction

**Fig. 5:**
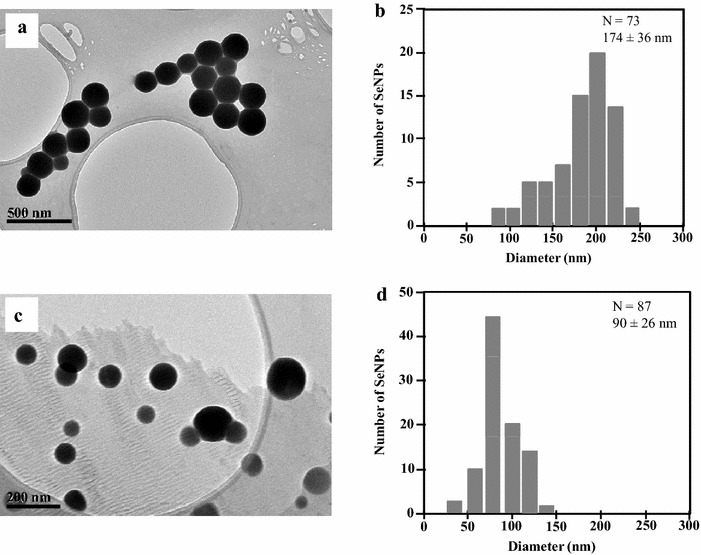
TEM (**a**, **c**) and size distribution (**b**, **d**) of SeNPs produced by *Azoarcus* resting cells under aerobic (**a**, **b**) or anaerobic (**c**, **d**) conditions

The use of whole bacteria or bacterial enzymes as biocatalysts is an attractive economical and green alternative to the large scale chemical synthesis of SeNPs [43, 44]. Furthermore, the biological synthesis allows the production of highly regular spherical SeNPs that cannot be obtained with chemical synthetic methods [43]. Therefore, it is interesting to search for biological systems that allow the synthesis of SeNPs under different operation conditions, e.g., in the presence or absence of oxygen, by using growing or resting cells, etc. In this sense, we have shown above that *Azoarcus* cells are tolerant to selenite when they grow anaerobically, triggering the synthesis of SeNPs when they reach the stationary-phase of growth. To check whether the reduction of selenite could be also performed in the presence of oxygen, *Azoarcus* cells were anaerobically grown and then collected in anaerobic conditions and resuspended in a solution containing 1 mM selenite. Whereas half of the resting cells solution was incubated in anaerobiosis, the other half was incubated under aerobic conditions. After 48 h, both cell solutions began to develop the orange color characteristic of the SeNPs. TEM analysis showed spherical nanoparticles whose average size was 174 ± 36 nm in aerobic (Fig. 5a, b) and 90 ± 26 nm in anaerobic (Fig. 5c, d) resting cells conditions. The SeNPs obtained from anaerobic resting cells showed similar size to that of SeNPs produced with cells that were cultivated anaerobically in the presence of selenite (Fig. 2e). Thus, these results indicate that the selenite reduction and formation of SeNPs by anaerobically grown *Azoarcus* cells occurs both, in the absence or in the presence of oxygen, and they suggest that the selenite reduction mechanism does not need to be induced by growing the cells in the presence of selenite. Moreover, these data demonstrate that the biosynthesis of SeNPs can be engineered as a resting cell process by using CIB whole cell biocatalysts that can be produced easily in the absence of selenite and then eventually used at high cell densities for an efficient bioconversion of selenite to SeNPs.

**Fig. 6:**
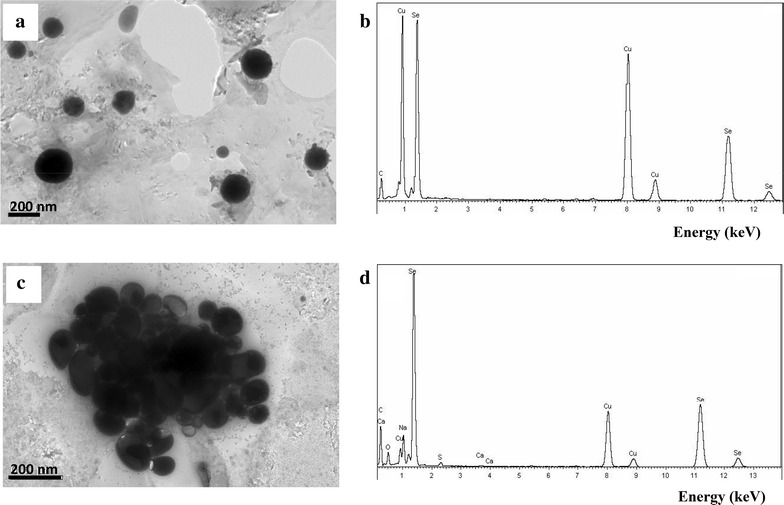
TEM and EDX of the SeNPs produced by *Azoarcus* sp. CIB cellular extracts (**a**, **b**) and by chemical reduction (**c**, **d**)

Next, it was explored whether SeNPs could be also synthesized by using cellular extracts. Addition of 1 mM selenite to the cellular extract and further aerobic incubation for 24 h at 30 °C revealed the appearance of the typical orange color due to selenite reduction. It is worth noting that whereas the in vitro chemical synthesis of SeNPs from selenite in the presence of glutathione produced mostly unstructured amorphous aggregates of metallic selenium (Fig. 6c, d), the biosynthesis of SeNPs with cellular extracts from *Azoarcus* generated spherical entities of average size 141 ± 37 nm (Fig. 6a; Additional file 4: Figure S4). EDX analysis showed that the electron-dense spherical particles presented the specific selenium absorption peak (Fig. 6b). These results demonstrate that selenite reduction and SeNPs formation can also be accomplished by developing a CIB-derived cell free process under aerobic conditions.

These results allow us to conclude that *Azoarcus* sp. CIB is a bacterium with potential for the development of cost-effective and environmental sustainable anaerobic/aerobic processes for the production of SeNPs. A comparison of some features of *Azoarcus* sp. CIB cultures grown in the presence of selenite showed similarities with those previously reported for other bacterial cultures (Additional file 5: Table S1). Control of the nanoparticle size and microbial metabolism involved in the nanomanufacturing will be analyzed in the next future.

## Conclusions

In this work, strain CIB was shown to be a bacterium capable to transform selenite (Na_2_SeO_3_) to Se (0) and generate SeNPs. A molecular mechanism of selenite resistance for the CIB strain growing under anaerobic conditions and based on the existence of an uncharacterized energy-dependent exporter of selenite out of cell that is functional only at the exponential phase of growth, has been proposed. In this sense, we present here an alternative to chemical methods based on the use of CIB as a promising biocatalyst, either as whole cells or cellular extract systems, for the anaerobic and/or aerobic green synthesis of spherical SeNPs of great interest in many biotechnological applications. On the other hand, chemical detoxification of metal and metalloid polluted sites has proven to be very expensive and often results in secondary effects in the environments. In contrast, microbiological methods generate nanoparticles that are regarded as safe, cost-effective and environment-friendly processes [20, 45]. The ability of strain CIB to produce SeNPs, together with the fact that this strain degrades different toxic aromatic hydrocarbons and it can colonize rice as an endophyte [25], opens the prospect to use this bacterium in general bioremediation strategies as well as in specific phytoremediation and cereal cultivation on selenium contaminated soils.

## Methods

### Strains and growth conditions

*Azoarcus* sp. CIB is deposited in the Spanish type culture collection (CECT#5669). *Azoarcus* sp. strain CIB was grown on MA basal medium (22; composed of the following, per liter of distilled water: 0.33 g of KH_2_PO_4_, 1.2 g of Na_2_HPO_4_, 0.11 g of NH_4_Cl, 0.1 g of MgSO_4_ × 7H_2_O, 0.04 g of CaCl_2_ [pH 7.5]) supplemented with trace elements [stock solution, 100×; 1.5 g of nitrilotriacetic acid, 3 g of MgSO_4_ × 7H_2_O, 0.5 g of MnSO_4_ × 2H_2_O, 1 g of NaCl, 0.1 g of FeSO_4_ × 7H_2_O, 0.18 g of CoSO_4_ × 7H_2_O, 0.1 g of CaCl_2_ × 2H_2_O, 0.18 g of ZnSO_4_ × 7H_2_O, 0.01 g of CuSO_4_ × 5H_2_O, 0.02 g of KAl(SO_4_)_2_ × 12H_2_O, 0.01 g of H_3_BO_3_, 0.01 g of Na_2_MoO × 2H_2_O, 0.025 g of NiCl_2_ × 6H_2_O, and 0.3 mg of Na_2_SeO_3_ × 5H_2_O (pH 6.5) per liter of deionized water], vitamin solution [stock solution, 1000×; 20 mg of biotin, 20 mg of folic acid, 10 mg of pyridoxine–HCl, 50 mg of thiamine-HCl × 2H_2_O, 50 mg of riboflavin, 50 mg of nicotinic acid, 50 mg of calcium d-pantothenic acid, 50 mg of vitamin B12, and 50 mg of *p*-aminobenzoic acid per liter of distilled water], and 10 mM potassium nitrate. For anaerobic growth, fifteen milliliters of MC medium (MA basal medium plus trace elements, vitamins, and nitrate) was flushed with N_2_, and the bottles were sealed with rubber stoppers and aluminum crimp seals before being autoclaved. For anaerobic growth, 10 mM potassium nitrate was usually added as electron acceptor as it has been previously described [22]. The carbon sources and the bacterial inoculum were injected through the stopper with a syringe. The cultures were incubated at 30 °C without shaking. As carbon source, 0.2 % (w/v) pyruvate was added. Aerobic cultures were incubated in the same MC medium, without nitrate, at 30 °C with shaking.

The microbial respiration of nitrate was monitored by measuring the levels of nitrate and nitrite by a nitrate colorimetric test strips, i.e., a white color on the strip represents exhaustion, and a violet color means that nitrate/nitrite are still present (Merck MQuant^®^). Since nitrate at concentration higher than 10 mM is toxic to the cells, we added firstly 10 mM nitrate and when consumed another 10 mM nitrate was added.

The microbial growth and reduction of selenite was studied with *Azoarcus* cells cultured aerobically or anaerobically at 30 °C in MC medium with pyruvate 0.2 % (w/v) as only carbon source and supplemented with different concentrations of sodium selenite; the coloration of the culture was monitored along the growth. When indicated, the cultures were amended with 3 mM BSO or 40 mg/l DNP from the beginning of the growth. Since DNP was prepared in methanol, control samples were amended with the same volume of this solvent.

Starvation growth conditions were achieved limiting either (i) the electron donor by using standard concentrations of nitrate (10 mM) and limiting amounts of pyruvate (0.1 %), or (ii) the acceptor donor by using standard concentrations of pyruvate (0.2 %) and limiting amounts of nitrate (5 mM). To prevent the starvation conditions additional amounts of carbon (0.2 % pyruvate) and electron acceptor (10 mM) were added.

### Reduction of selenite and SeNPs production

Time course of selenite concentration in the culture samples was determined by either inductively coupled plasma optical emission spectrometry (ICP-OES) (Perkin Elmer Optima 2100 DV) [46] or by an adaptation of a colorimetric method previously described [47]. Briefly, 1 ml of bacterial culture was centrifuged for 60 min at 12,650*g* and then 600 μl of the supernatant was mixed with 300 μl of 4 M HCl and 600 μl of a solution of 1 M ascorbic acid. The reaction mix was incubated for 10 min at room temperature, and then the absorbance was measured at 500 nm. Values were interpolated in a standard selenite calibration curve. No change in selenite concentration or appearance of orange color was observed in MC medium containing 1 mM selenite and incubated anaerobically without cells for 7 days at 30 °C.

To determine which fraction of the cell culture is responsible of selenite reduction, we grew anaerobically *Azoarcus* cells up to the end of the exponential phase and then the culture was pelleted and the cells and the culture supernatants were collected separately. The cells were resuspended in an equal volume of saline solution and lysed by sonication obtaining the crude extract. One fraction of the culture supernatant and the crude extract were heated at 100 °C for 10 min whereas the other fraction was maintained at 4 °C. Total protein concentration in each fraction was determined by Bradford [48].

For the production of SeNPs with *Azoarcus* resting cells, cells were anaerobically grown in MC medium in the absence of selenite by using 0.2 % pyruvate as electron donor and 10 mM nitrate as electron acceptor. Cells were harvested at stationary phase (*A*_600_ of 0.8) by centrifugation for 20 min at 3800*g* in an anaerobic glovebox, washed twice with 50 mM HEPES buffer pH 7.5, and resuspended in the same HEPES solution. Half of the cell suspension was incubated at 30 °C for 48 h in the anaerobic glovebox after the addition of 1 mM of sodium selenite. The other half was supplemented with 1 mM of selenite and incubated at 30 °C for 48 h under aerobic conditions in Erlenmeyer flasks. Samples were recovered by centrifugation 2 min at 11,600*g* and prepared for TEM analyses (see below).

For the chemical production of SeNPs with glutathione, the procedure followed was an adaptation of the protocol established by Ramos and Webster [49]. After the addition of 2 ml of 100 mM glutathione to a 6 ml of a solution of 25 mM selenite, the medium was basified with 0.66 ml of 1 M NaOH and incubated at room temperature. Almost immediately an orange color developed suggesting the fast reduction of the selenite to Se (0).

For the production of SeNPs with bacterial cellular extracts, cells were incubated anaerobically until the end of the exponential phase of growth (*A*_600_ of 0.8), washed and resuspended in sterile 0.9 % NaCl solution. The cellular extracts were produced by sonication (4 times for 30 s), and 600 μl of cellular extract was mixed with 1 mM sodium selenite. After aerobic incubation at 30 °C for 24 h, the orange color developed in the reaction assay was monitored, and the SeNPs characterized by TEM and EDX analyses.

### Characterization of SeNPs

For field emission Scanning Electron Microscopy (SEM), the samples were filtered through 0.2 μm pore-size filters and successively dehydrated with acetone/water mixtures of 30, 50 and 70 % acetone, respectively, and stored overnight at 4 °C in 90 % acetone. After critical-point drying, samples were coated with graphite and gold and examined with a JEOL JSM-6330 F microscope.

For transmission electron microscopy (TEM) analysis, the samples were prepared by placing drops of the cell culture onto carbon-coated copper grids and allowing the solvent to evaporate. Ultrathin sections of each sample were cut with a Reicher Ultracut S Ultramicrotome (Vienna, Austria) fitted with a diamond knife.TEM observations were performed on a JEOL model JEM-2100 instrument operated at an accelerating voltage of 200 kV. The chemical composition of the SeNPs observed was determined by energy-dispersive X-ray spectroscopy (EDX) as previously described [47].

The size of nanoparticles was determined by using the Image J software [50].

## Additional files

10.1186/s12934-016-0510-y Time course of *Azoarcus* growth at different selenite concentration in anaerobic (A) and aerobic (B) conditions.
10.1186/s12934-016-0510-y Selenite reduction assays carried out in different cell fractions. The protein concentration of each fraction is indicated.
10.1186/s12934-016-0510-y Five days anaerobic growth of *Azoarcus* in the presence or absence of selenite and BSO.
10.1186/s12934-016-0510-y Size distribution of SeNPs produced by *Azoarcus* cellular extracts.
10.1186/s12934-016-0510-y Features of some bacterial cell cultures in the presence of selenite.

## Abbreviations

SeNPs: selenium nanoparticles
ICP-OES: inductively coupled plasma optical emission spectrometry
SEM: scanning electron microscopy
TEM: transmission electron microscopy
EDX: energy-dispersive X-ray spectroscopy
SAED: selected area electron diffraction
DNP: 2,4-dinitrophenol
BSO: buthionine sulfoximidine
