# Supplementary figures and images for: Biosynthesis of selenium nanoparticles by Azoarcus sp. CIB
Source: Microb Cell Fact. 2016 Jun 14;15:109. doi: 10.1186/s12934-016-0510-y (PMC4908764; doi:10.1186/s12934-016-0510-y)

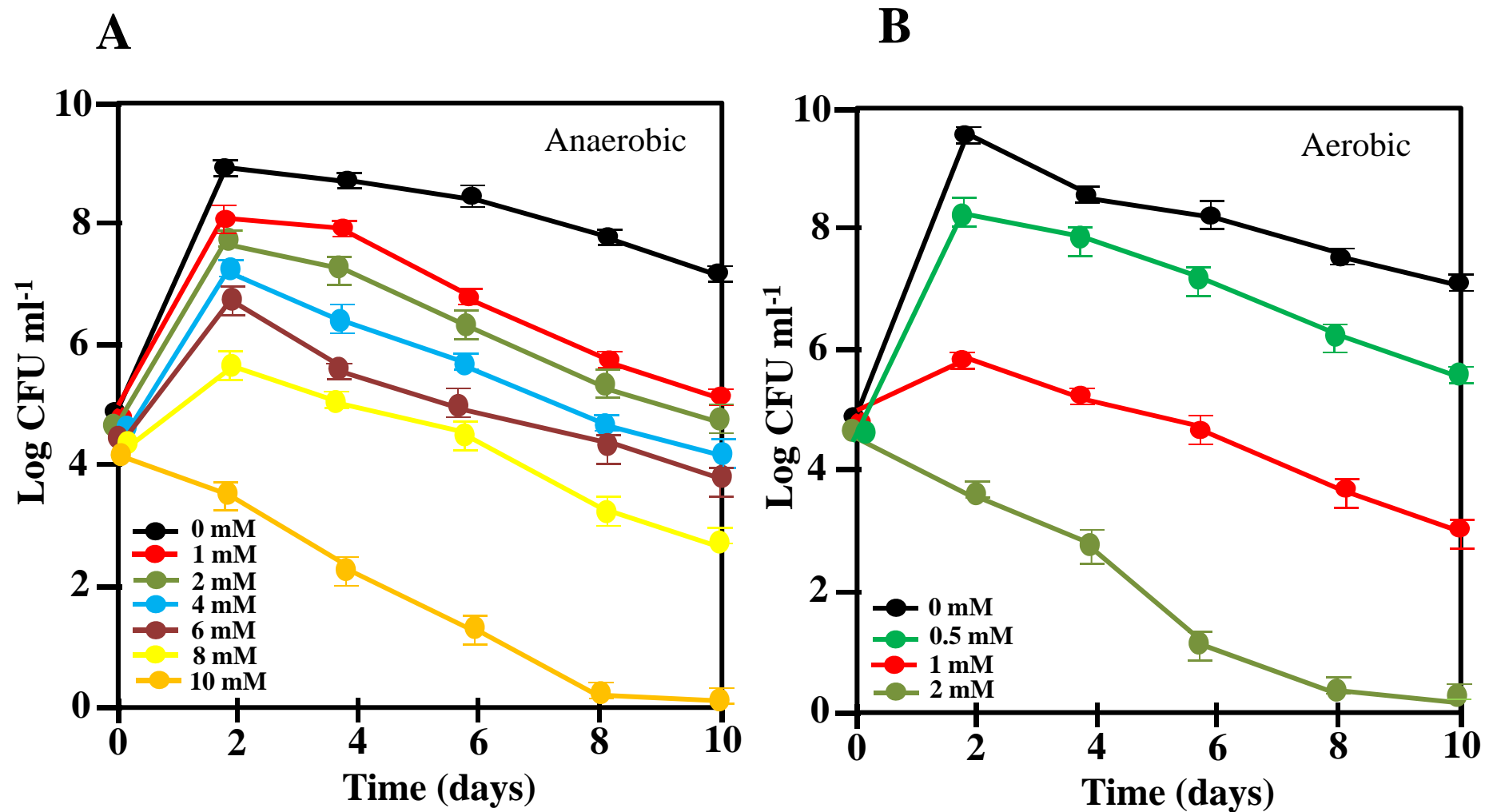

**Fig. S1**

Supplement: Supplementary file 1 — 10.1186/s12934-016-0510-y Time course of Azoarcus growth at different selenite concentration in anaerobic (A) and aerobic (B) conditions. [file 12934_2016_510_MOESM1_ESM.pdf]

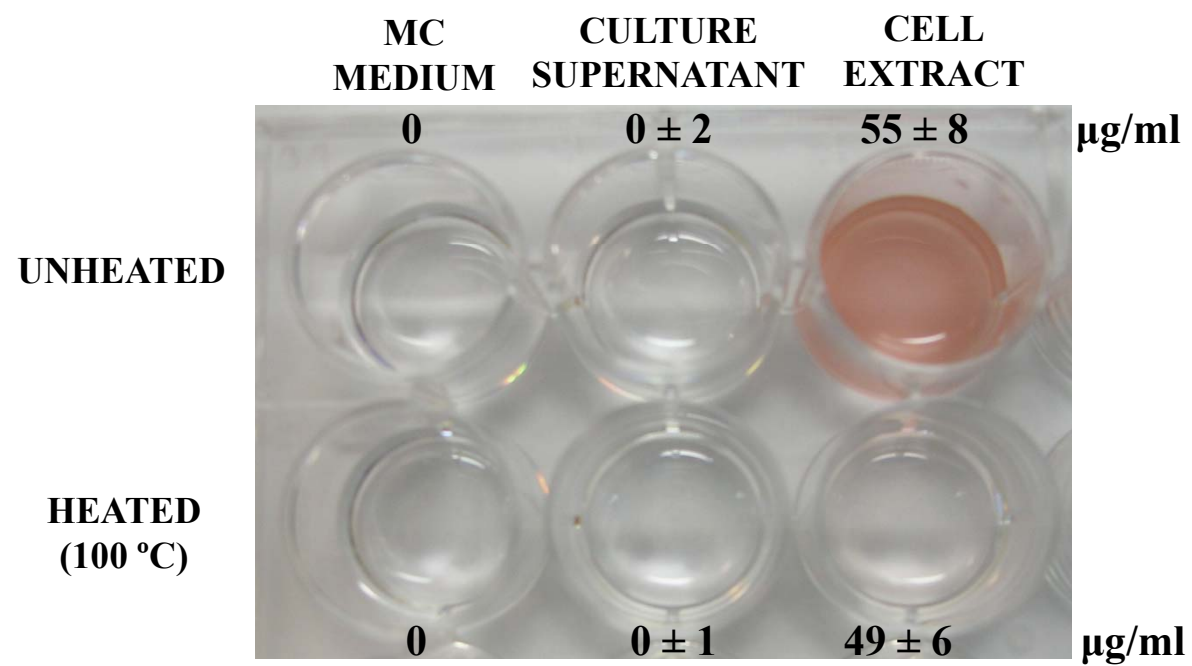

**Fig. S2**

Supplement: Supplementary file 2 — 10.1186/s12934-016-0510-y Selenite reduction assays carried out in different cell fractions. The protein concentration of each fraction is indicated. [file 12934_2016_510_MOESM2_ESM.pdf]

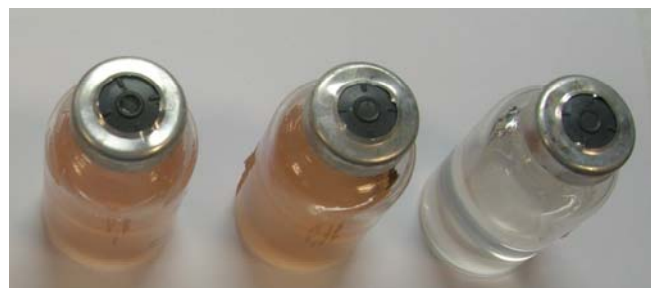

|                      |          |          |          |
|----------------------|----------|----------|----------|
| <b>BSO (mM)</b>      | <b>0</b> | <b>3</b> | <b>0</b> |
| <b>Selenite (mM)</b> | <b>1</b> | <b>1</b> | <b>0</b> |

**Fig. S3**

Supplement: Supplementary file 3 — 10.1186/s12934-016-0510-y Five days anaerobic growth of Azoarcus in the presence or absence of selenite and BSO. [file 12934_2016_510_MOESM3_ESM.pdf]

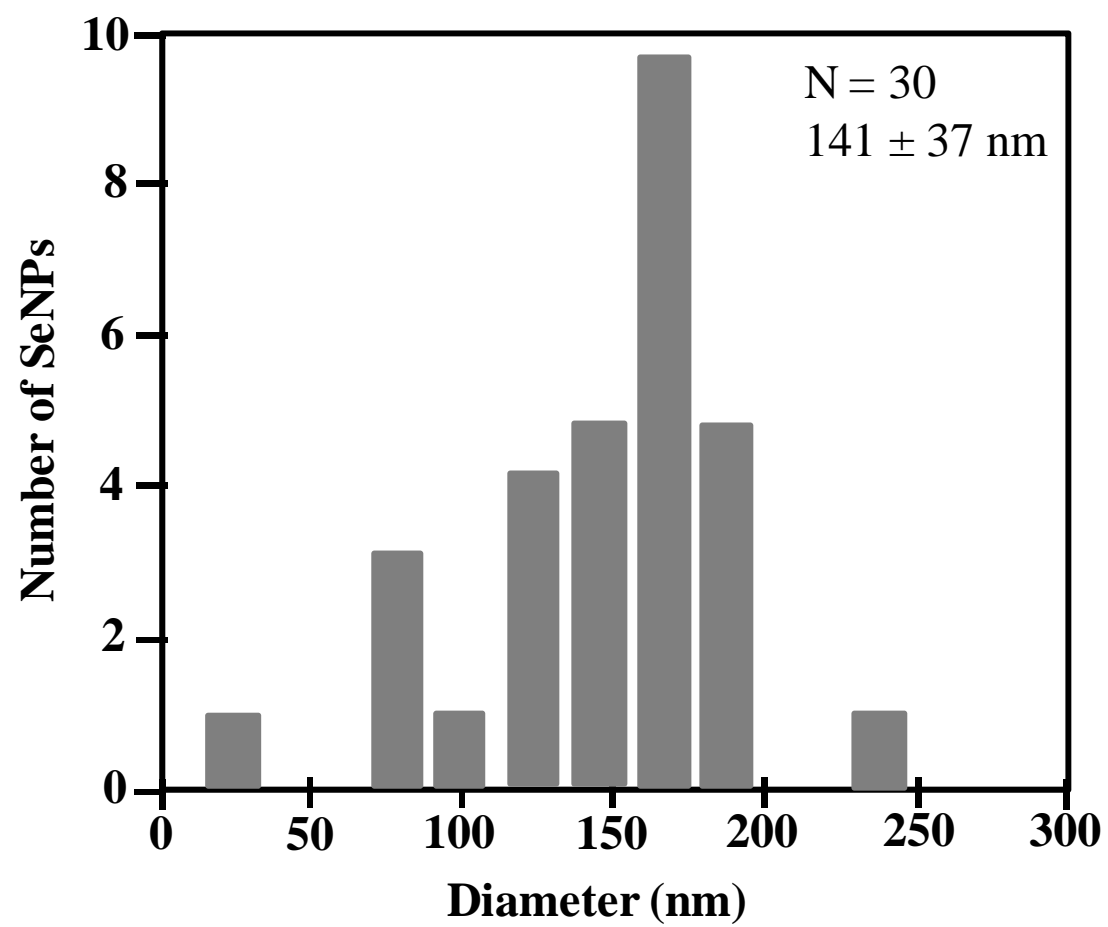

**Fig. S4**

Supplement: Supplementary file 4 — 10.1186/s12934-016-0510-y Size distribution of SeNPs produced by Azoarcus cellular extracts. [file 12934_2016_510_MOESM4_ESM.pdf]
