# Supplementary material for: Biosynthesis of selenium nanoparticles by Azoarcus sp. CIB
Source: Microb Cell Fact. 2016 Jun 14;15:109. doi: 10.1186/s12934-016-0510-y (PMC4908764; doi:10.1186/s12934-016-0510-y)
Supplement: Supplementary file 5 — 10.1186/s12934-016-0510-y Features of some bacterial cell cultures in the presence of selenite. [file 12934_2016_510_MOESM5_ESM.docx]

**Table S1. Feature of some bacteria cell culture in the presence of selenite**

**Bacteria Resistance Conditions SeNPs size % Selenite transformation* Reference**

*Rhodospirillum rubrum* 1.5 mM Anaerobic ND 100% in 80 h 30

*Enterobacter cloacae* SLD1a1 ND Anaerobic ND 83% in 60 h 45

*Rhodopseudomonas palustri*s SeTE02 8 mM Anaerobic 80-200 nm 100% in 216 h 31

*Bacillus cereus* >10 mM Aerobic 150-200 nm 100% in 24 h 46

*Stenotrophomonas maltophilia* SelTE02 >5mM Aerobic 100-300 nm 100% in 48 h 47

*Pseudomonas moraviesis* 120 mM Aerobic ND 100% in 48 h 48

*Comamonas testosteroni S44*  150 mM Aerobic 100-200 nm 60% in 25 h 49

*Bacillus mycoides* Sel TE01 25 mM Aerobic 50-400 nm 100% in 24 h 50

*Synechococcus leopoliensis* >5 mM Aerobic 174-390 nm 20% in 216 h 51

*Shewanella* sp. HN-41 >1 mM Anaerobic 11-20 nm 60% in 70h 52

*Azoarcus* sp. CIB 8 mM Anaerobic 88±40 nm 70% in 144 h This work

*: The percentage of transformation depends on the initial number of cells in the culture, initial amount of selenite, and time of sampling. Details are shown in the references. ND: not determined.

References:

30. Kessi J, Ramuz M, Wehrli E, Spycher M, Bachofen R. Reduction of selenite and detoxification of elemental selenium by the phototrophic bacterium *Rhodospirillum rubrum*. Appl Environ Microbiol. 1999;65:4734-4740.

31. Li B, Liu N, Li Y, Jing W, Fan J, Li D, Zhang L, et al. Reduction of selenite to red elemental selenium by *Rhodopseudomonas palustris* strain N. PLoS ONE 2014;9:e95955.

45. Yee N, Kobayashi DY. Molecular genetics of selenate reduction by *Enterobacter* *cloacae* SLD1a-1 Adv Appl Microbiol. 2008;64:107-123

46. Dhanjal S, Cameotra SS. Aerobic biogénesis of selenium nanospheres by *Bacillus cereus* isolated from coalmine soil. Microbial Cell Fact. 2010;9:52.

47. Lampis S, Zonaro E, Bertolini C, Cecconi D, Monti F, et al. Selenite biotransformation and detoxification by *Stenotrophomonas maltophilia* SeITE02: Novel clues on the route to bacterial biogenesis of selenium nanoparticles. J. Hazard Mater. 2016;S0304-3894:30162-30165.

48. Staicu LC, Ackerson CJ, Cornelis P, Ye L, Berendsen RL et al. Pseudomonas moraviensis subsp. stanleyae, a bacterial endophyte of hyperaccumulator *Stanleya pinnata*, is capable of efficient selenite reduction to elemental selenium under aerobic conditions. J. Appl. Microbiol. 2015;119:400-410.

49. Zheng S, Su J, Wang L, Yao R, Wang D, et al. Selenite reduction by the obligate aerobic bacterium Comamonas testosteroni S44 isolated from a metal-contaminated soil. BMC Microbiol. 2014;14:204.

50. Lampis S, Zonaro E, Bertolini C, Bernardi P, Butler C, et al. Delayed formation of zero-valent selenium nanoparticles by Bacillus mycoides SelTE01 as a consequence of selenite reduction under aerobic conditions. Microbial Cell Fact. 2014;13:35.

51. Hnain A, Brooks J, Lefebvre DD. The synthesis of elemental selenium particles by *Synechococcus leopoliensis*. Appl. Microbiol. Biotechnol. 2013;97:10511-10519.

52. Tam K, Ho CT, Lee J-H, Lai M, Chang CH et al. Growth mechanism of amorphous selenium nanoparticles synthesized by *Shewanella* sp. HN-41. Biosci. Biotechnol. Biochem. 2010;74:696-700.
